# Numerical Analysis on Transverse Splicing Structure for the Widening of a Long Multi-Span Highway Concrete Continuous Box Girder Bridge

**DOI:** 10.3390/ma15196805

**Published:** 2022-09-30

**Authors:** Wenqing Wu, Hui Zhang, Zheng Liu, Yunpeng Wang

**Affiliations:** 1 School of Transportation, Southeast University, Nanjing 210096, China; 2 Quality and Safety Technology Department, Jiangsu Provincial Transportation Engineering Construction Bureau, Nanjing 210004, China

**Keywords:** bridge widening, transverse stress, corrugated sheet plate (CSP), shrinkage and creep, settlement difference, parameter optimization

## Abstract

For the bridge widening of long multi-span highway concrete continuous box girder with a conventional splicing structure, due to the large longitudinal difference deformation by concrete shrinkage and creep between the existing and new ones, the widened structure will have an overlarge bending deformation after widening, especially an obvious transverse deformation at the end of girder, which will lead to structural damage to the newly widened structure. To effectively absorb the difference deformation mentioned above, this study proposes a novel transverse splicing structure based on the folding effect of a corrugated steel plate (CSP) (hereinafter referred to as “the CSP splicing structure”). Then, a finite element structural analysis was performed on the mechanical properties of the widened structure with the CSP splicing structure, and compared to those of a widened structure adopting the conventional concrete splicing mode, to clarify the transverse load transferring mechanism of the structure. Finally, by conducting a sensitivity analysis on CSP thickness, corrugation length, splicing stitch width, and other structural parameters, a sound parameter combination scheme was put forward. According to the research results, to ensure effective utilization of the CSP folding effect, the corrugation pattern direction of CSP should be set as horizontal, and the wave angle as the degree of 90°. In addition, it mitigated the transverse tensile stress to effectively avoid concrete cracking feasibility on the top flange of the box girder at the end of the girder. This study offers a feasible way of avoiding the structural damage produced by an excess transverse deformation at the end of the girder after bridge widening of a long multi-span concrete continuous box girder.

## 1. Introduction

Economic development and population growth have put tremendous pressure on transportation services. Therefore, many existing highway bridges cannot meet the current increased traffic demands. Widening existing highway bridges not only requires less time to improve their capacity but also saves investment. A common way to do this is to build a new bridge side by side with the existing one and structurally connect the two bridge decks with an in situ concrete stitching slab. At present, the huge demand for bridge widening mainly comes from highway bridges, not railway bridges.

**Figure 1:**
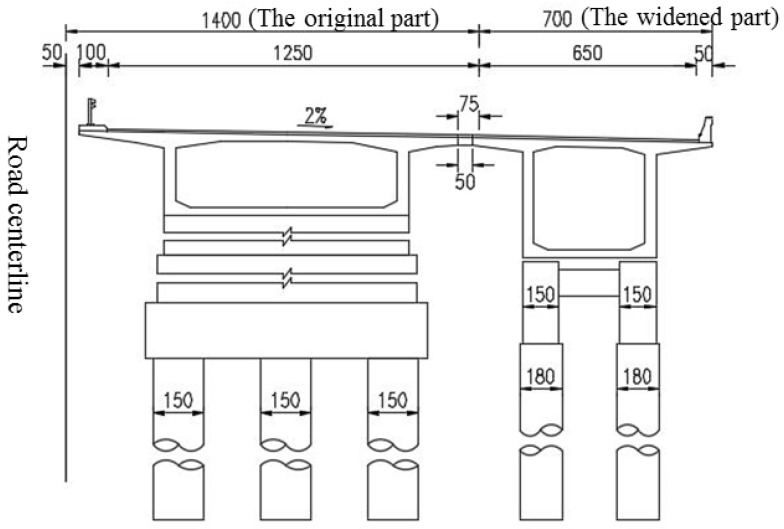
Transverse widening mode of connection superstructures but disconnection substructures.

Currently, integral widening, in which the new section of the structure is made continuous with the existing one, is attractive because it avoids the complication of a movement joint that would often be below a running carriageway [1]. Under this scheme, the substructures of new and existing bridges are separated from each other, while their superstructures are transversely spliced together through structural connections and collectively subjected to overall stress, as shown in Figure 1.

**Figure 2:**
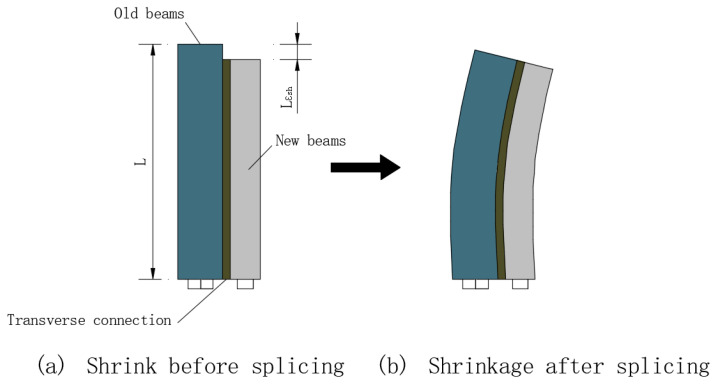
Transverse deformation of widening structure induced by concrete shrinkage.

The interactions between the new and existing bridges are incurred by their different completion times, producing restraint stresses in the new and existing structures. For instance, the significant differences in their different completion times between the new and existing bridges would result in a series of deformation differences between them, including the longitudinal shrinkage and creep deformation of concrete, vertical foundation settlement, and so forth [1]. To be specific, the longitudinal concrete shrinkage and creep deformation of the new bridge are restrained by the existing bridge, which not only produces secondary structural internal forces but also causes overall transverse bending deformation in the widened bridge [2,3,4,5,6], as shown in Figure 2.

**Figure 3:**
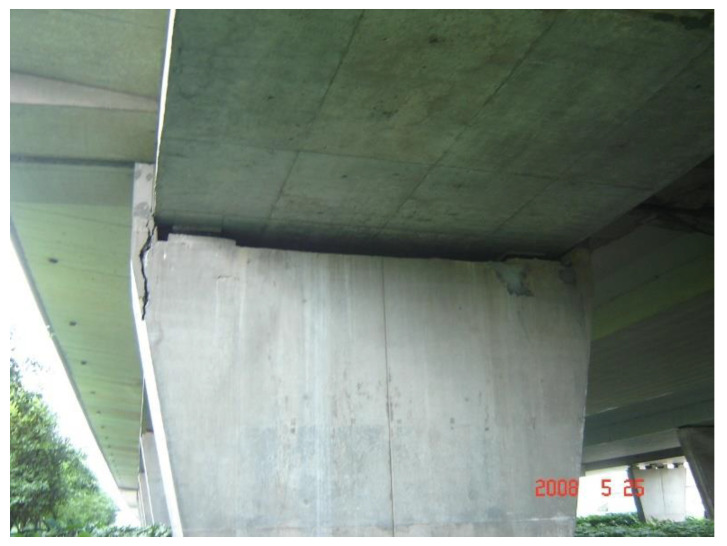
Anti-knock block failure because of transverse deformation of the girders.

A practical engineering case described in the literature [3] involves a prestressed concrete continuous box girder bridge with a multi-span length of 38.5 + 65 + 38.5 m, a total length of 142 m, and a net single-deck width of 12.5 m. In the present widening mode, a prestressed concrete continuous box girder with the same span layout was placed on one side of the existing bridge. After a deck width increase of 8.25 m, the single-deck bridge had a full deck width of 20.75 m. Within one year after splicing widening, the structure showed an obvious transverse bending deformation, characterized by outward transverse deformation at both ends of the continuous girder. Due to the huge deformation (as large as 45 mm), the end of the box girder seriously squeezed the external anti-knock block, cracking the anti-knock block and causing serious deformation and partial failure of supports, as illustrated in Figure 3. With the increasing length of the continuous girder bridge, this structural deformation increased significantly. This poses a huge challenge to the transverse splicing of long-unit concrete continuous girder bridges [7]. Therefore, analyzing the transverse effect of the structure induced by concrete shrinkage and creep after multi-span bridge widening is very valuable.

To mitigate the adverse effect because of the different completion times of the new and existing bridges, the method of delayed splicing of the new and existing bridges for a period of six months is usually adopted worldwide to reduce the adverse effects of differences in deformation between the new and existing bridges on the widened structure [8,9,10]. However, for the widened structures of multi-span long-unit bridges, a delay of six months alone does not fundamentally solve the problem [11]. Shi et al. [7] analyzed the effects of long-term loads on the mechanical performance of three widened long-unit prestressed concrete girder bridges. Their results indicated that, under four long-term loads (i.e., concrete shrinkage and creep, temperature gradient, and overall temperature rise and decline), long-unit bridges would produce huge transverse deformation, and the maximum deformations occurred on the abutments or the transition piers of one-unit continuous girder bridges, as shown in Figure 2. Moreover, the transverse displacement caused by concrete shrinkage accounted for 69% of the total transverse displacement produced by the four long-term loads, indicating that concrete shrinkage is the dominant factor affecting the transverse deformation of a widened long-unit bridge. Wen [6] analyzed an example of a prestressed concrete box-girder bridge widening. According to the calculated results, longitudinal tensile stress at the flange of the new girder can reach 2.84 MPa due to shrinkage and creep effects of the new box girder after 10 years, which may lead to concrete cracks. Chen et al. [12] proposed an “indirect splicing” method for the splicing widening of new and existing bridges, in which a longitudinal spliced segment is set by intervals along the widened bridge to reduce the integrity of the widened bridge after splicing; however, in that case, it would be difficult to coordinate the deformation of the new and existing structures in the unspliced segments. Thus, in-depth studies are still needed to understand the stress mechanism of transverse internal force transfer.

On the whole, as far as the widening structures of long concrete continuous girder bridges are concerned, the study on the interaction mechanism between the new and existing bridges and the countermeasures is still in the start-up stage, and the lack of related research results has severely hindered the further development of bridge-widening technologies and should be urgently addressed.

## 2. Research Background

In this study, a bridge of Beijing-Shanghai Expressway was taken as the engineering background. The bridge is a prestressed concrete continuous box girder bridge with a variable cross-section and a total length of 214 m, including a four-span of 40 + 2 × 67 + 40 m.

**Figure 4:**
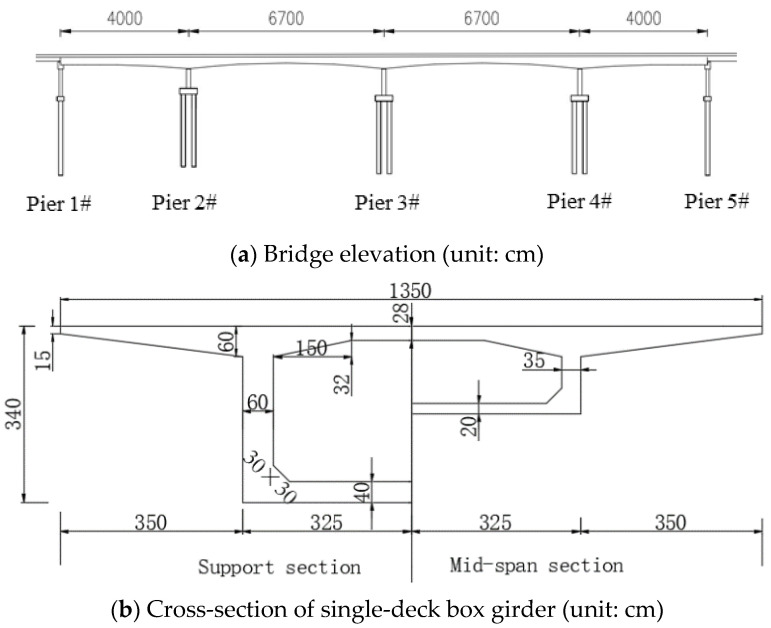
General layout of a bridge.

**Figure 5:**
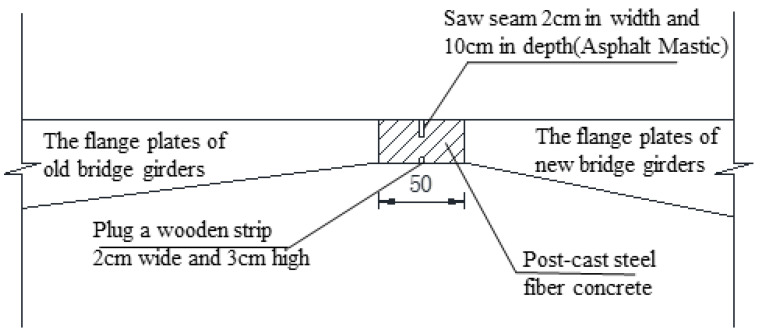
Schematic diagram of the traditional transverse splicing structure.

The bridge deck is divided into 4-lane double-way, and the single-way box girder uses a single-box single-cell section, as shown in Figure 4. The existing bridge was widened by constructing a new box girder by one side of the existing bridge, and then the superstructure of the new and existing box girder was connected by a concrete stitching slab, while substructures are disconnected. Only the flange plates of the new and existing bridge girders are transversely spliced but not the transverse diaphragms. Figure 5 shows a traditional transverse splicing structure. According to the relevant literature [3,13], if the transverse splicing structure shown in Figure 5 is adopted because of the longitudinal deformation differences between the new and existing bridges in concrete shrinkage and creep, the widened structure will produce an obvious transverse deformation, and the maximum transverse deformation at both ends of the continuous girder will reach 68 mm, severely impairing its structural safety.

To effectively deal with the differences between the new and existing bridges in the longitudinal shrinkage and creep deformation of concrete after widening, this study proposes a novel transverse splicing structure based on the folding effect of a corrugated steel plate (CSP). By investigating its technical feasibility and force transferring mechanism and exploring its reasonable structural parameters, this study aims to offer a new solution to the splicing widening of long multi-span concrete continuous bridges.

## 3. Effectiveness of CSP Splicing Structure

**Figure 6:**
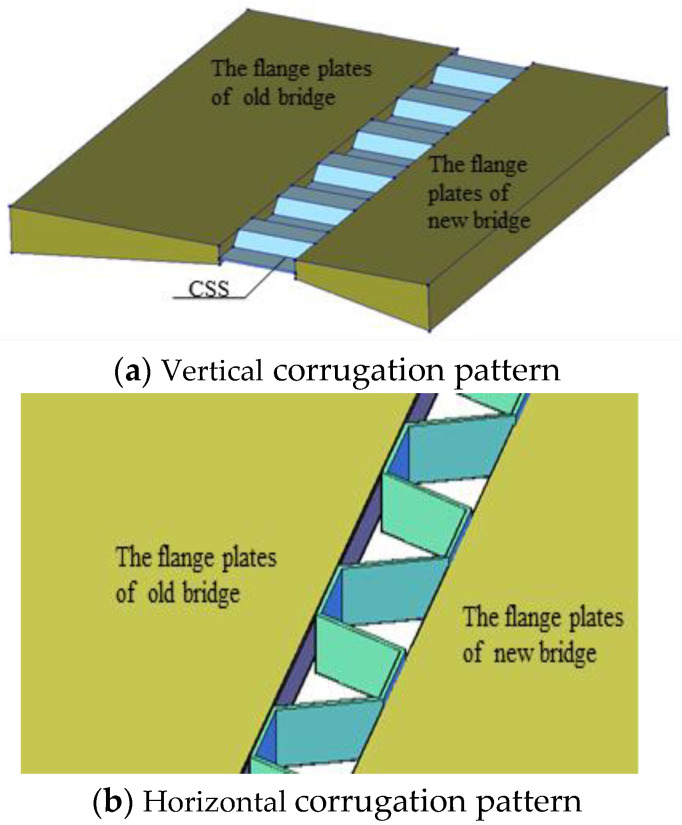
Schematic diagram of a comparison of CSP connection schemes.

**Figure 7:**
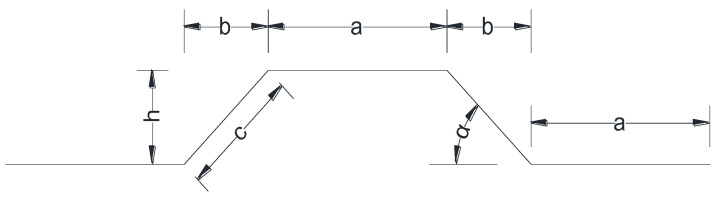
Schematic diagram of the wave angle of the corrugated steel plate. (a: length of straight segment, b: projection length of slant segment, c: length of slant segment, h: wave height, α: wave Angle).

CSP is widely used in continuous box girder bridges with corrugated steel webs, mainly by virtue of its sound longitudinal folding effect. On the basis of such a longitudinal folding effect, two possible splicing structures were put forward, as shown in Figure 6. To comparatively study different structural layout schemes, two transverse connection schemes are proposed according to the different corrugation pattern directions of CSP: (i) vertical corrugation pattern, under which CSP is vertically corrugated, and (ii) horizontal one, where CSP is horizontally corrugated. Depending on the specific wave angle of the corrugated steel plate, each type is further divided into two forms, i.e., right angle and acute angle according to the size of the wave angle (see Figure 7).

Before building the FE model, the mechanical properties of steel and concrete are listed in Table 1. All standard values of the cube compressive strength ***f_cu_***, the axial compressive strength ***f_ck_***, and tensile strength ***f_tk_***, the elastic modulus **E_c_** of C50 concrete can be determined according to the reference [13,14]. All standard values of the yielding strength ***f_y_***, the ultimate strength ***f_u_*** and the elastic modulus **E_s_** of Q345 steel plate concrete can be determined according to the reference [14].

**Figure 8:**
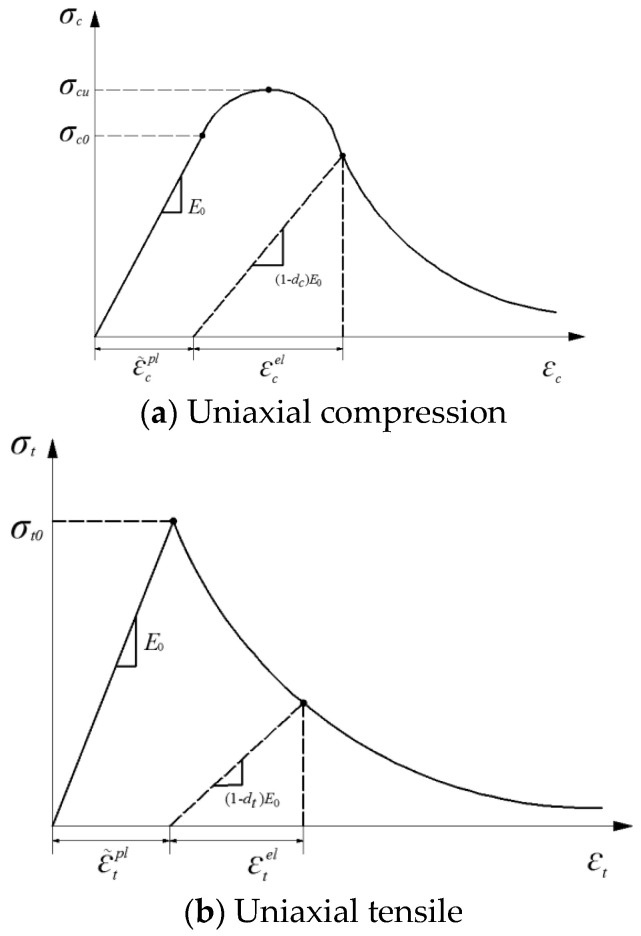
Stress−strain relation of concrete.

**Figure 9:**
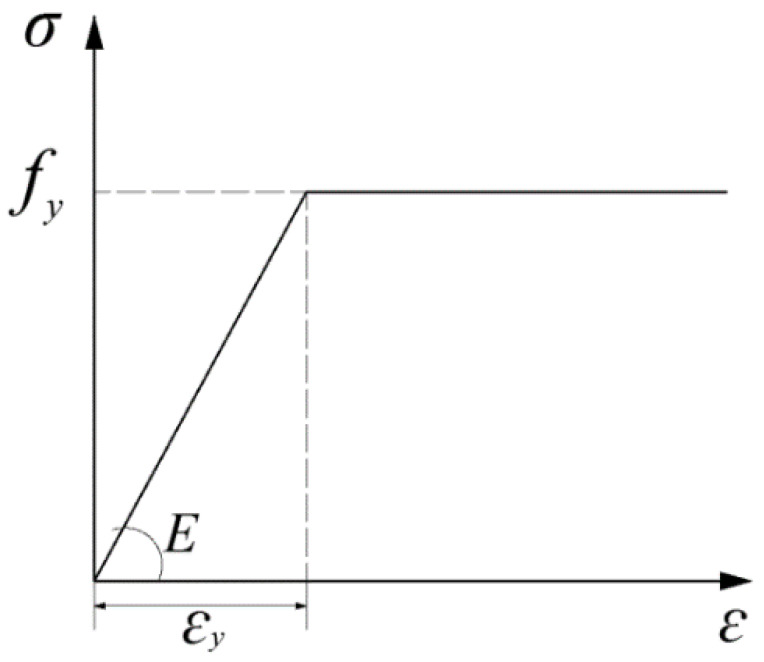
Stress−strain relation of steel.

The stress-strain relation of concrete damage constitutive in this paper is the concrete stress-strain relation suggested in Appendix C of the Code for Design of concrete Structures of China [14] (as shown in Figure 8 and Figure 9).

Shrinkage and creep coefficients were adopted according to the CEB-FIP model (CEB, 1990), and the relative humidity of the ambient environment was set at 60% [15].

**Figure 10:**
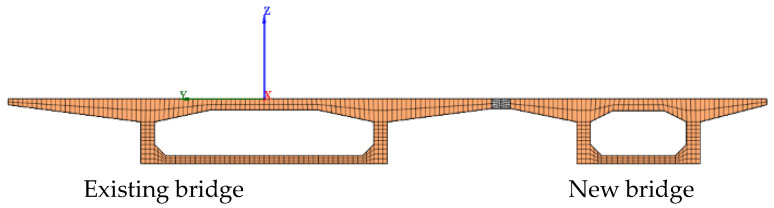
Schematic diagram of meshing cross-section of overall bridge model.

On this basis, two overall 3D finite element models were built for bridge widening. The overall dimensions of the two models followed the design drawings of the bridge under investigation in this study. Considering the different stress characteristics and thickness of structural members, two types of elements (3D solid elements and plate shell elements) were used for modeling: new and existing concrete box girders are both made of C50 concrete and simulated using eight-node hexahedral elements; CSP uses Q345 steel that is 10 mm in thickness and was simulated using four-node plane elements. Figure 10 shows a schematic diagram of the meshing cross-section of a full-bridge finite element model. As for the connection between the steel plate and the flange plate of the existing and new box girder, in the overall model, it is simplified that the bolt acts as a connection. The connection action was modeled using spring elements that had longitudinal capabilities in 1-D, 2-D, or 3-D applications [16]. The longitudinal spring-damper is a uniaxial tension-compression element with up to three degrees of freedom at each node: translations in the nodal x, y, and z directions.

**Figure 11:**
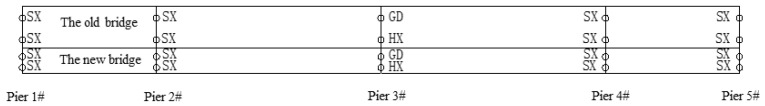
Support layout of the model.

Figure 11 shows various pivot support restraint conditions, where SX, ZX, HX, and GD denote bilaterally slidable support, longitudinally slidable support, transversely slidable support, and fixed support, respectively.

The load case considers only the differences in concrete shrinkage and creep between the new and existing bridges, for which the structure produces transverse deformation, as shown in Figure 2. Through the established finite element analysis model, the paper first assumed that the existing bridge had been working for 10 years and then considered the creep and shrinkage deformation of the new and old bridge for 10 years of joint work after the completion of bridge widening [5,7]. This paper focuses on the longitudinal shrinkage and creep deformation difference between old and new structures, as well as the stress and deformation state of the widened structure under the effect of the deformation difference mentioned above.

Table 2 provides the transverse deformation values at the end of the girder produced by the widened bridge under the action of the differences in deformation between the new and existing bridges in concrete shrinkage and creep under different CSP connection schemes. Recent studies have shown that, for a bridge widened using the traditional transverse splicing structure, the girder-end transverse deformation can reach 68 mm under the same working conditions [3].

The data listed in Table 2 lead to the following facts:

(1) When the “CSP connection with vertical corrugation pattern” shown in Figure 6a was adopted, neither the right-angle steel plate nor the acute-angle steel plate could effectively reduce transverse deformation at the end of the girder under the action of shrinkage and creep deformation difference of the new and existing bridges. In this case, the displacement value was nearly the same as that in cases where traditional splicing structures were used, indicating that the corrugated steel plate hardly exerts a “folding effect.” This analysis suggests that under the action of the differences in deformation between the new and existing bridges in the longitudinal shrinkage and creep deformation of concrete, CSP is in a state of longitudinal eccentric compression instead of uniform compression, indicating that CSP can exert a “folding effect” only through the local non-uniform extrusion deformation of the structure; however, the small width of the splicing stitch seriously restricts the “folding effect” of CSP.

(2) Under the “CSP connection scheme with horizontal corrugation pattern,” it was possible to effectively reduce the transverse displacement at the end of the girder of the widened bridge only when a right-angle CSP is adopted. According to the analysis, when there was a longitudinal deformation difference between the new and existing box girder flange plates, the CSP set between them produced longitudinal compression, inevitably resulting in transverse expansion of the CSP structure. However, such transverse expansion was resisted by both the new and existing flange plates on both sides, making it impossible for the CSP to bring its “folding effect” into play. In contrast, CSP bent at a right angle (“right-angle CSP” for short) avoided this problem and adapted well to the differences between the new and existing bridges in the longitudinal shrinkage and creep deformation of concrete. The splicing structure with a horizontal CSP for right-angle bending is discussed later.

**Figure 12:**
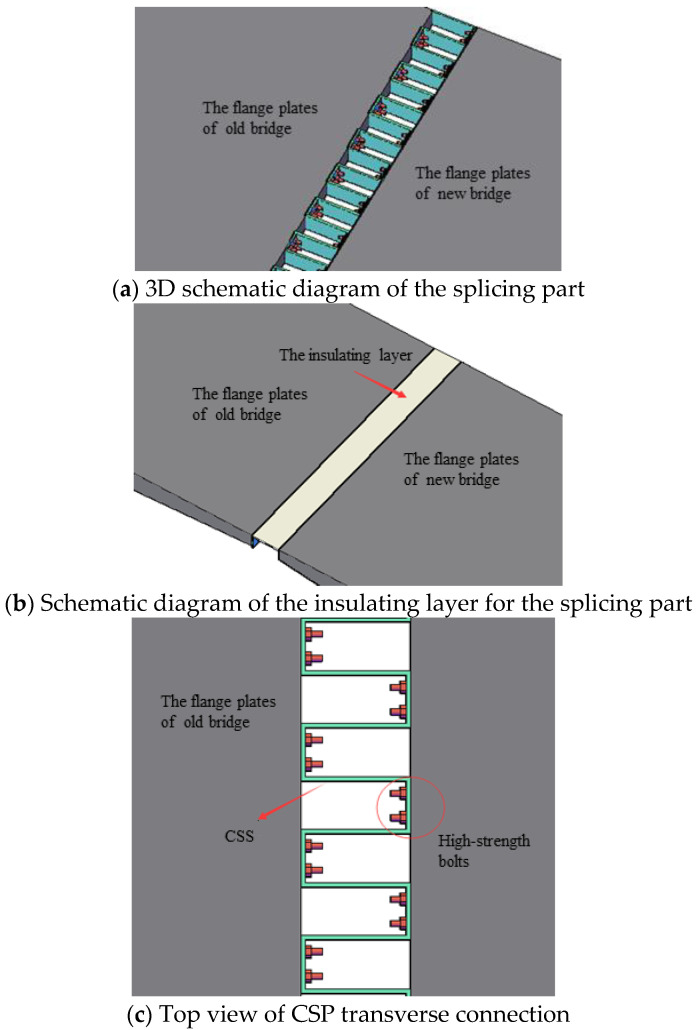
Schematic diagram of the horizontal CSP splicing structure.

Based on the analysis of the above two types of CSP layout schemes, in this study, we proposed a horizontal CSP splicing structure bent at a right angle, which is applicable to the widening of a long-unit concrete continuous box girder bridge (hereinafter referred to as “the CSP splicing structure”), as shown in Figure 12. The invention patent was granted [17] (Chinese patent number: 201710545690.2).

In the constitution of the structure, the first right-angle CSP is arranged continuously along the longitudinal splicing stitch, and the corrugation pattern direction was set as horizontal; the peaks and valleys of CSP were connected to the new and existing box girder flange plates using high-strength bolts, thus creating a transverse splicing structure. Next, a thin sheet steel isolated layer was prepared at the top of the CSP structure to bear the bridge deck pavement. Finally, a new bridge deck was paved on the entire spliced segment to form a flat bridge deck. The isolated layer can be welded together with the top surface of the box girder on one side and set free on the other side. In this way, it will neither restrict the longitudinal deformation of CSP nor cause it to slip out of service.

## 4. Analysis of the Mechanical Behavior of a CSP Transverse Splicing Structure

### 4.1. Analysis of the Working Mechanism

**Figure 13:**
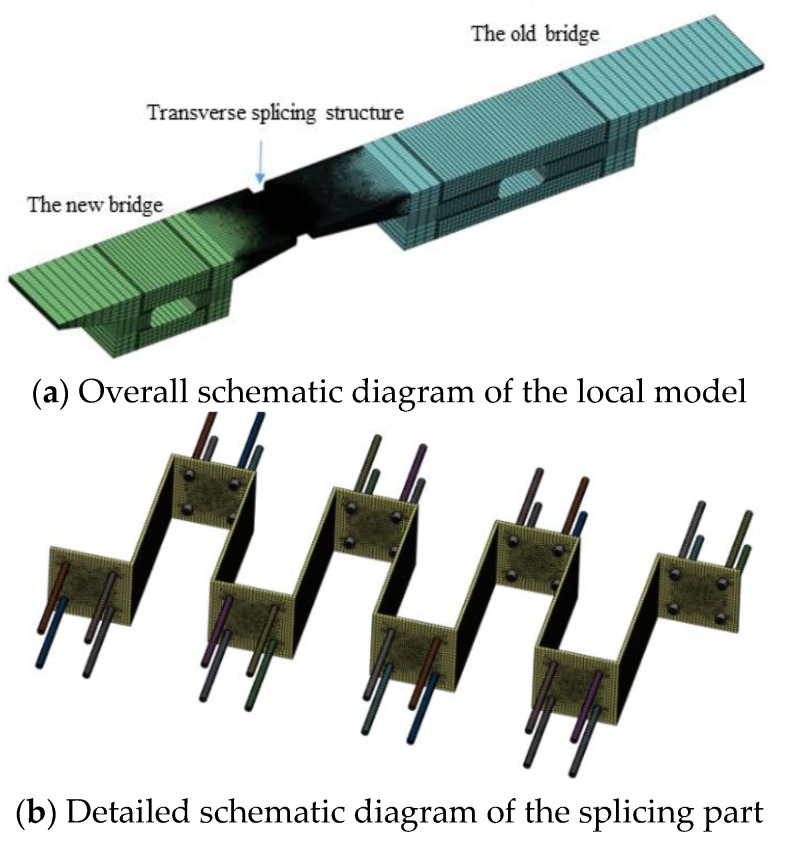
Schematic diagram of the local finite element model.

In order to illustrate the working mechanism of the new splicing jointing structure, an analysis is conducted by taking a local structure with a length of 2 m longitudinally around the end support of the girder and building a refined local FE model, as shown in Figure 13a. The CSP splicing structure uses a right-angle CSP with a thickness of 10 mm, a corrugation height of 0.5 m, and a corrugation length of 0.5 m to analyze the deformation state of the novel splicing structure and its stress change rules under the action of the differences between the new and existing bridges in concrete shrinkage and creep.

In the local refined finite element model, the new and existing box girder concrete, steel plate, and bolts were all simulated using solid elements, and the elements within the splicing part were refined again. The model has a total of 496,455 elements, and its boundary conditions are simulated as a hinging in the four corners at the bottom of the box girder. To be specific, CSP is connected to the new and existing flange plates by using four bolts of 20 mm in diameter and 15 d in anchorage depth, as shown in Figure 13b. The bonding slip between the internal screw and the concrete is not considered in this model, and the sides of the steel plate are connected to the sides of the existing and new concrete flange plate using a spring element with compression only.

**Figure 14:**
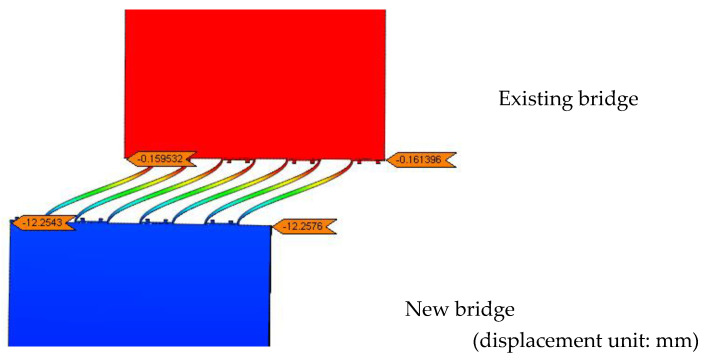
Deformation of CSP after application of longitudinal forced displacement on the new bridge.

To effectively apply the effect of structural deformation difference, all the nodes on the end cross-section within the range of the new bridge are rigidly connected to the centroid node of the cross-section, followed by applying a longitudinal forced displacement at the mass centroid to simulate the longitudinal shrinkage and creep displacement difference between the existing and new bridges. According to the global finite element model shown in Figure 10, the maximum value of the longitudinal forced displacement difference was 13 mm. Figure 14 shows a schematic diagram of the deformation at the splicing part of the new and existing box girder after applying a longitudinal forced displacement to the new bridge.

Figure 14 shows that the deformation of transverse steel plates is dominated by S-shaped bending deformation, which allows the splicing structure to easily absorb the large longitudinal dislocation deformation. This deformation characteristic ensures that the longitudinal deformation difference between the new and existing box girders is almost entirely borne by CSP, resulting in a very small longitudinal restraining effect of the existing bridge on the new bridge. The above deformation characteristics reveal the working mechanism of the splicing structure with a CSP that can adapt well to the longitudinal shrinkage and creep deformation differences between the new and existing bridges.

### 4.2. Effects of Different Splicing Modes on the Overall Stress Characteristics of the Widening Structure

**Figure 15:**
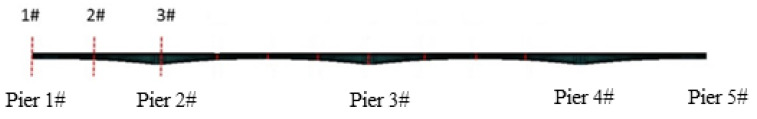
Schematic diagram of a longitudinal control section.

To investigate the differences in stress characteristics under the traditional splicing mode and the CSP splicing mode, two finite element models were built to stimulate two types of transverse splicing structures and then to analyze the widening structure. Three key sections were selected, as shown in Figure 15.

The combination value of actions comprises dead weight, prestress, shrinkage creep, foundation post-construction settlement difference, lane load, and so forth. Figure 14 shows the transverse stress variation of the top fiber of the box girder bridge deck under a combination action case on the 1#–3# sections under the action combination. Specifically, the foundation post-construction settlement difference refers to the difference between the new and existing bridges in the foundation settlement deformation after transverse splicing. Generally, it is assumed that the settlement deformation of the existing bridge foundation is 0, and that produced by the new bridge is equal to the post-construction settlement difference. For the bridge under investigation in this study, the foundation post-construction settlement difference was set at 5 mm [13,18,19].

**Figure 16:**
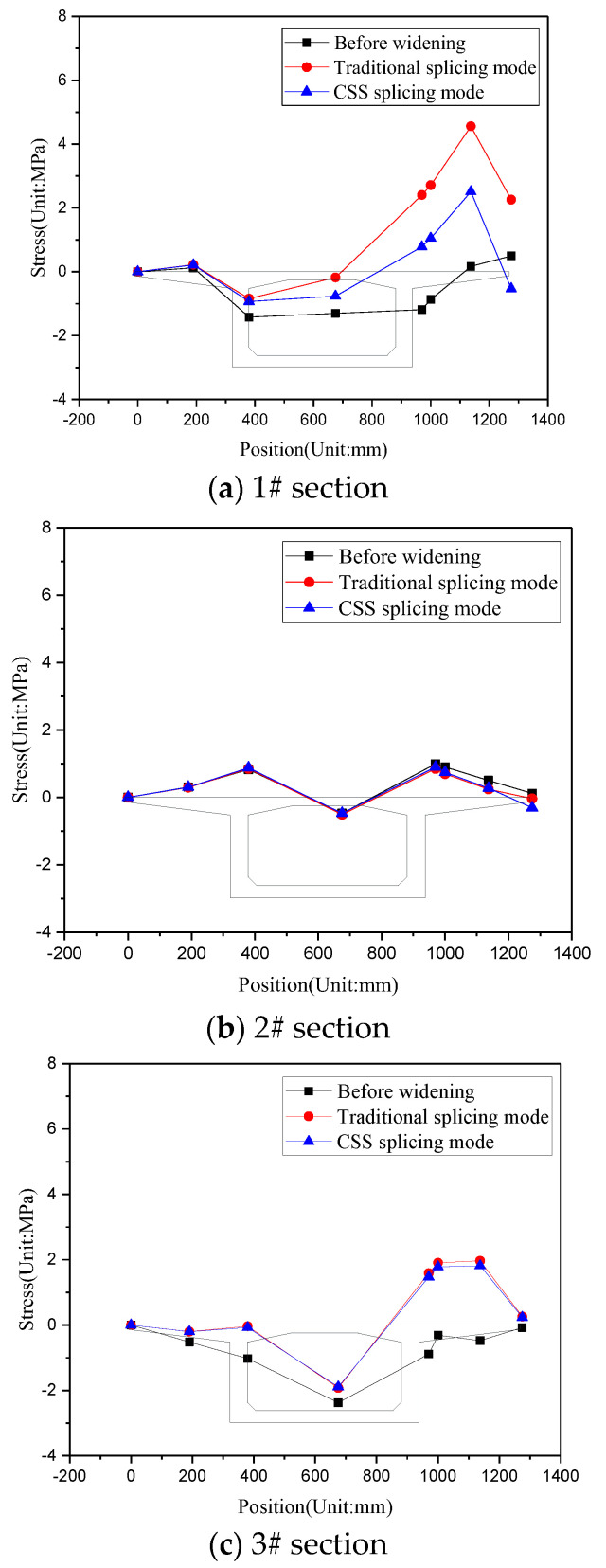
Transverse stress variation of the top fiber of the box girder bridge deck.

Figure 16 shows that, (1) on 1# and 3# support sections, the transverse tensile stress at the top slab of the existing-bridge box girder increased significantly but remained unchanged basically in the 2# section of the side span. This analysis reveals that the foundation post-construction settlement difference mainly occurs in the support sections but is very small in the mid-span section. This is the reason for the obvious increase in the transverse stress of the support section. (2) Splicing widening has the largest adverse effect on the girder-end (1#) section of the existing bridge. Under traditional hinged splicing, the transverse tensile stress of the inner flange plate of the box girder reaches 4.557 MPa, and the maximum tensile stress occurs at the upper middle edge of the flange plate, far exceeding the standard tensile strength of C50 concrete (2.65 MPa). The inner flange plate of the existing bridge will be cracked in a large area, requiring effective reinforcement measures. In contrast, when CSP splicing is used for splicing widening, the maximum tensile stress on the inner flange plate of the existing bridge box girder is 2.510 MPa, which has a great advantage over the traditional splicing method. Moreover, the maximum principal tensile stress of the corrugated steel plate under comprehensive working conditions was 207.541 MPA, not exceeding the yield strength of the Q345 steel plate.

## 5. Parameters and Dimensional Design of CSP Splicing Structures

The selected analysis parameters included CSP thickness, CSP wavelength, and splicing stitch width. Given that the differences between the new and existing bridges in concrete shrinkage and creep and the foundation post-construction settlement are the main factors affecting the stress performance of the splicing widening structure, the parameter sensitivity analysis was performed mainly under the two action cases:

Action case 1: The action of the differences between the new and existing bridges in concrete shrinkage and creep deformation;

Action case 2: The action of the difference between the new and existing bridges in foundation post-construction settlement.

The analysis results of the three parameters are listed in Table 2, Table 3 and Table 4. The data in the table were analyzed as follows:

(1) Table 3 shows that with increasing CSP thickness, the transverse displacement at the end of the girder increases significantly, while other structural responses, such as the transverse tensile stress on the concrete flange plate and the tensile stress on the steel sheet, are not sensitive to this change. This shows that when the thickness of the corrugated steel plate increases, its splicing stiffness will increase significantly, significantly decreasing its ability to absorb the longitudinal concrete shrinkage and creep of new and existing bridges. Therefore, the thickness of the steel plate should be relatively small and should be 10 mm or 15 mm.

(2) Table 4 shows that when the width of the splicing stitch increases from 0.5 m to 1.0 m, the transverse displacement value of the girder-end section decreases significantly, while other structural responses are not sensitive to such change. This indicates that increasing splicing stitch width decreases the splicing stiffness of CSP, which further weakens the restraining effect of the existing bridge on the new bridge and significantly decreases transverse displacement. However, the width of the splicing stitch should not be too large. When the width reaches 1.0 m, the deflection difference between the new and existing flange plates, induced by the foundation settlement of the new bridge on the support section, increases significantly and is not conducive to road safety. Based on the relevant literature [19], it is appropriate to control the relative deflection difference of the flange end of the new and old bridges within 2 mm, so the width of the patchwork joints should not be too large. The optimum width of the splicing stitch was set at 0.5 m or 0.75 m.

(3) Table 5 shows that when corrugation length increases from 0.5 m to 1.0 m, the transverse displacement value of the girder-end section decreases. Apparently, an increased corrugation length decreased the splicing stiffness of sheet steel and significantly reduced transverse displacement. However, when the corrugation length reaches 1.0 m, a high degree of concrete stress concentration occurred within the range of the corrugated steel plates and the concrete flange plates of the new and existing box girders connected thereto, and is detrimental to the deformation coordination between the new and existing box girders. Thus, the corrugation length of CSP is still recommended to be 0.5 m.

Based on the analysis results of the sensitivity of parameters, in this study, for the sake of achieving a better splicing effect, two parameter value schemes are proposed, i.e., Scheme 1: right-angle CSP with a thickness of 10 mm, a splicing stitch width of 0.5 m, and a corrugation length of 0.5 m; Scheme 2: right-angle CSP with a thickness of 15 mm, a splicing stitch width of 0.75 m, and a corrugation length of 0.5 m. The advantages and disadvantages of the two parameter optimization schemes were further analyzed, as detailed in Table 6.

According to Table 6, the maximum transverse displacement values of girder-end section of both schemes are both 20 mm. However, based on the stress states of the new and existing box girder flange plates and CSP, Scheme 2 is considered a better scheme.

## 6. Conclusions

In conclusion, this study successfully demonstrated the scheme design and working mechanism of a CSP splicing structure based on a case study of a bridge of Beijing-Shanghai Expressway. The sensitivity of the relevant parameters was analyzed, and a better scheme design was put forward for this type of splicing structure. The results of this study are as follows:

(1) When the “CSP connection scheme with a vertical corrugation pattern” is adopted, the corrugated steel plate with a right angle or an acute angle cannot effectively reduce lateral deformation at the end of the widened bridge girder, indicating that the corrugated steel plate almost does not exert a “folding effect.” When the “CSP connection scheme with a horizontal corrugation pattern” is adopted, only when the wave of right angle in the steel plate is adopted can the corrugated steel plate effectively exert the “folding effect,” and reduce the transverse deformation at the end of girder under the action of concrete shrinkage and creep deformation difference between the new and existing bridge. Based on the analysis results, the CSP splicing structure waved at the right angle is proposed and can be effectively applied to the transverse splicing widening of long multi-span concrete continuous box girder bridges.

(2) When the CSP splicing structure is adopted as the widening stitch structure for the long multi-span concrete continuous box girder bridge, the maximum transverse deformation at the end of girder is about 20 mm, about 1/3 of that under the traditional splicing scheme. This indicates that the CSP splicing structure can adapt well to the action of the differences between the new and existing bridges in concrete shrinkage and creep deformation.

(3) The working mechanism of the right-angle CSP is explained as follows. Due to the longitudinal deformation difference between the new and existing box girders, the deformation of the sheet steel is dominated by S-shaped bending deformation; the splicing structure has a strong ability to absorb longitudinal displacement, and the longitudinal restraint effect of the existing bridge on the new bridge is very small. As a result, the CSP splicing structure can adapt well to the differences between the new and existing bridges in the longitudinal shrinkage and creep deformation of concrete.

(4) Under the combined action, when traditional splicing is adopted, the transverse tensile stress on the top flange plate at the end of girder of the existing bridge reaches 4.557 MPa, far exceeding the standard tensile strength of C50 concrete, making it necessary to take effective anti-cracking and reinforcement measures. In contrast, when the CSP splicing structure is adopted, both the maximum tensile stress on the top flange plate of the box girder of the existing bridge and the maximum principal tensile stress on the CSP are within the safe range, indicating that the comprehensive stress performance of the CSP splicing structure is obviously superior to that of the traditional splicing structures.

(5) Through investigating the rules of the effects of CSP thickness, corrugation length, splicing stitch width, and other structural parameters on the stress characteristics of the widening structure, a sound parameter combination scheme was successfully established for the CSP splicing structure. The optimum CSP thickness, splicing stitch width, corrugation length, and waving angle were found to be 15 mm, 75 cm, 0.5 m, and right angle, respectively.

## Figures and Tables

**Table 1:** Specifcation value of material mechanical strength/MPa.

Material	Category	Specifcation Value
C50 concrete	***f_ck_***	32.4
	***f_cu_***	57.5
	***f_tk_***	2.64
	**E_c_**	345,000
Q345 Steel plate	***f_y_***	345
	***f_u_***	510
	**E_s_**	200,000

**Table 2:** Transverse deformation at the girder end under different CSP connection schemes (mm).

Connection Scheme	Bending Angle	Existing Bridge	New Bridge
Vertical corrugation pattern	Right angle	−67.77	−67.76
	Acute angle	−67.31	−67.34
Horizontal corrugation pattern	Right angle	−20.54	−20.28
	Acute angle	−66.98	−67.06

**Table 3:** Sensitivity analysis of CSP thickness.

Action Case	Index	CSP Thickness/mm		
		10	15	20
1	Transverse displacement at the end of girder/mm	20	39	52
	Tensile stress on the existing bridge flange plate at girder end/MPa	0.65	0.85	1.04
	Tensile stress on new-bridge flange plate at the end of the girder/MPa	1.78	1.68	1.57
	Principal tensile stress on the CSP/MPa	197.94	197.15	173.98
2	Deflection difference between the new and existing flange plates at side support/mm	1.289	1.225	1.189
	Maximum tensile stress on existing bridge flange plate at side support/MPa	4.59	4.61	4.60
	Maximum tensile stress on the new-bridge flange plate at the side support/MPa	4.25	4.49	4.67
	Principal tensile stress on CSP/MPa	87.06	66.05	54.33

**Table 4:** Sensitivity analysis of the splicing stitch width.

Load Case	Index	Splicing Stitch Width/m		
		0.5	0.75	1.0
1	Transverse displacement at girder end/mm	20	9	5
	Tensile stress on existing bridge flange plate at girder end/MPa	0.65	0.52	0.45
	Tensile stress on new-bridge flange plate at girder end/MPa	1.78	1.81	1.81
	Principal tensile stress on CSP/MPa	197.94	137.13	95.49
2	Deflection difference between the new and existing flange plates at side support/mm	1.289	1.834	2.321
	Maximum tensile stress on the existing bridge flange plate at the side support/MPa	4.59	4.06	3.57
	Maximum tensile stress on the new-bridge flange plate at the side support/MPa	4.25	3.71	3.22
	Principal tensile stress on the CSP/MPa	87.06	97.98	88.08

**Table 5:** Sensitivity analysis of corrugation length.

Load Case	Index	Corrugation Length/m	
		0.5	1.0
1	Transverse displacement at girder end/mm	20	8.5
	Tensile stress on the existing bridge flange plate at the end of the girder/MPa	0.65	0.49
	Tensile stress on the new-bridge flange plate at the end of the girder/MPa	1.78	1.82
	Principal tensile stress on the CSP/MPa	197.94	161.63
2	Deflection difference between the new and existing flange plates at side support/mm	0.868	1.027
	Maximum tensile stress on the existing bridge flange plate at the side support/MPa	4.63	4.68
	Maximum tensile stress on the new-bridge flange plate at the side pivot/MPa	3.96	7.92
	Principal tensile stress on the CSP/MPa	87.06	126.29

**Table 6:** Comparison of the two parameter optimization schemes.

LOAD Case	Index	Scheme 1	Scheme 2
1	Transverse displacement at the end of girder/mm	20	20
	Tensile stress on the existing bridge flange plate at girder end/MPa	0.65	0.63
	Tensile stress on the new-bridge flange plate at the end of girder/MPa	1.78	1.75
	Principal tensile stress on CSP/MPa	197.94	165.32
2	Deflection difference between the new and existing flange plates at the side support/mm	1.289	1.727
	Maximum tensile stress on the existing bridge flange plate at the side support/MPa	4.59	4.14
	Maximum tensile stress on the new-bridge flange plate at the side support/MPa	4.25	3.97
	Principal tensile stress on the CSP/MPa	87.06	74.58

## Data Availability

Not applicable.
